# Expression levels of induced sputum IL-8 and IL-10 and drug intervention effects in patients with acute exacerbated COPD complicated with chronic cor pulmonale at high altitude

**DOI:** 10.3892/etm.2013.1192

**Published:** 2013-07-01

**Authors:** ENZHI FENG, RONGHUA WAN, SHENGYUE YANG, ZIQIANG YAN, SHAOLIN WANG, WEI HE, YING ZHANG, HE YIN, ZONGRU CHEN, RUINIAN LIU

**Affiliations:** 1 Center of Respiratory Medicine, The Fourth Hospital of PLA, Lanzhou Command, Xining, Qinghai 810007, P.R. China; 2 Department of Cardiovascular Surgery, Heart Center, General Hospital of Ningxia Medical University, Yinchuan, Ningxia 750004, P.R. China

**Keywords:** interleukin-8, interleukin-10, corticosteroid, β_2_-adrenoceptor agonist, chronic obstructive pulmonary disease, chronic cor pulmonale

## Abstract

The aim of this study was to assess the expression levels of induced sputum interleukin (IL)-8 and IL-10 levels in patients with acute exacerbated chronic obstructive pulmonary disease (AECOPD) complicated with chronic cor pulmonale (CCP) at high altitude, and to evaluate the intervention effects of an inhaled corticosteroid (ICS) and a β_2_-adrenoceptor agonist in this disease. A total of 186 patients with AECOPD complicated with CCP were randomly divided into three groups, with 62 cases in each. With regard to the two treatment groups, group A was treated with salmeterol/fluticasone (50 μg/250 μg, respectively) by airway inhalation twice daily, while group B received budesonide (1 mg) as a spray inhalation, twice daily. The routine treatment group (group C) received only routine treatment. The levels of IL-8 and IL-10 in the induced sputum and the predicted percentage of forced expiratory volume in one second (FEV_1%pred_), partial pressure of oxygen in arterial blood (PaO_2_) and partial pressure of carbon dioxide in arterial blood (PaCO_2_) were examined on admission and at a stable stage two weeks following treatment. Forty healthy volunteers served as a control group (group D). Compared with group D values, the IL-8 induced sputum level and the PaCO_2_ were significantly increased, while the level of IL-10, FEV_1%pred_ and the PaO_2_ were markedly decreased in the three COPD groups prior to treatment. Following treatment, the induced sputum IL-8 level and the PaCO_2_ were significantly decreased, while the induced sputum IL-10 level, FEV_1%pred_ and the PaO_2_ were markedly increased in the three treatment groups compared with the values pre-therapy (all P<0.01). The post-treatment parameters were significantly different among the three groups (P<0.01). The results indicate that IL-8 and IL-10 are involved in the airway inflammation of AECOPD complicated by CCP. Treatment with an ICS was demonstrated to be a successful method of reducing the local expression of IL-8 and increasing the local expression of IL-10; however, ICS combined with a long-acting β_2_-adrenoceptor agonist (LABA) was more effective than the sole administration of ICS in patients with AECOPD complicated by CCP at high altitude.

## Introduction

Chronic obstructive pulmonary disease (COPD) is a common respiratory disease characterized by an airflow limitation that is not fully reversible. The airflow limitation is usually progressive and is correlated with an abnormal inflammatory response of the lungs to noxious particles or gases. The disease may be prevented and treated; however, it develops progressively. In recent years, COPD morbidity and mortality have increased rapidly, and it has been revealed that the global incidence of COPD has reached up to 10% (1). There are 600 million patients worldwide, and COPD is fatal for 2.75 million people each year (2). While in 1990 COPD was the fourth leading cause of global mortality, it has been predicted that it is likely to rise to the third by 2020. It is one of the five most prevalent diseases (3,4). Therefore, there is an urgent requirement for further studies into the pathogenesis and treatments of COPD. COPD is a chronic airway inflammation involving numerous cytokines and mediators of inflammation. The pathology of disease involves chronic and non-specific inflammation of the airway, lung parenchyma and pulmonary vessels and remodeling of the airway and vessel wall caused by repair. This leads to a progressive, persistent restriction to the air flow and a reduction in the lung function. A characteristic of acute inflammation is the high expression of inflammatory factors (5,6). The inflammation in COPD is a complex reactive procedure, involving a multi-pathophysiological development, including tissue lesions, airway remodeling and high levels of mucous secretion. Interleukin-8 (IL-8), a selective neutrophil chemotactic factor, is a cytokine that promotes the inflammatory response, whereas IL-10, an anti-inflammatory cytokine, is important in the regulation of the inflammatory reaction (7,8). It has been demonstrated that the level of IL-8 is increased and that of IL-10 is decreased significantly in the airways of patients with COPD in flatland areas (9–11). However, there has been limited investigation into the effects of the disease at altitude. Furthermore, the expression levels of IL-8 and IL-10 in the induced sputum of patients with acute exacerbated COPD (AECOPD) complicated by chronic cor pulmonale (CCP) have not yet been fully elucidated. Calverley *et al*(12) observed that an inhaled corticosteroid (ICS), combined with a long-acting β_2_-adrenoceptor agonist (LABA) significantly suppressed inflammatory markers in patients with COPD in flatland areas, and notably improved clinical parameters. However, the correlation between the anti-inflammatory effects and the clinical improvement was not revealed. Furthermore, it was not determined whether IL-8 and IL-10 exhibited any regulatory action.

In the present study, it was hypothesized that, at high altitude, IL-8 and IL-10 in the induced sputum of patients with AECOPD complicated by CCP are likely to be expressed abnormally and involved in airway inflammation. It was further suggested that treatment with ICS/LABA combination inhalers was likely to be able to regulate this effect. Therefore the expression levels of IL-8 and IL-10 in the induced sputum of patients with AECOPD complicated by CCP at high altitude were investigated and the intervention effects of ICS/LABA combination inhalers were observed. The results of the study were used to elucidate the mechanism of the inflammatory reaction in the airways of patients with AECOPD complicated by CCP at altitude, to investigate the novel therapeutic approach and to provide a foundation for its use as an anti-inflammatory therapy.

## Subjects and methods

### Patients

A total of 186 patients with AECOPD complicated by CCP at altitude (2,260~3,500 m) who were hospitalized in the Department of Respiratory Medicine at The Fourth Hospital of PLA (Xining, China) from June 2010 to September 2012 were selected for this study. The diagnosis of COPD was consistent with the standard Guidelines for the Diagnosis and Treatment of COPD (2007 Revised Edition), established by the respiratory branch of the Chinese Medical Association (13), while CCP was diagnosed according to the corresponding Chinese diagnostic criteria (14). The patients were randomly divided into three groups, with 62 cases in each. Two of the three groups, groups A and B, were treatment groups. The first treatment group, group A, comprised 36 males and 26 females, aged from 46 to 77 years (mean age, 58.6±7.5 years), while in group B there were 33 males and 29 females, aged from 48 to 77 years (mean age, 58.1±7.4 years). The routine treatment group (group C) comprised 35 males and 27 females, aged from 46 to 74 years (mean age, 57.9±7.2 years), while the control group (group D) consisted of 26 male and 14 female healthy volunteers, aged from 50 to 72 years (mean age, 58.1±7.0 years). The age and gender ratio in the three groups demonstrated no statistical significances (all P>0.05). This study was conducted in accordance with the Declaration of Helsinki and with approval from the Ethics Committee of The Fourth Hospital of PLA. Written informed consent was obtained from all participants.

### Medication program

Routine treatment, such as anti-infective agents, expectorants and aminophylline, was identical in the three groups. In addition to the routine treatment, the patients in group A were treated with ICS/LABA combination inhalers, containing salmeterol/fluticasone (50 μg/250 μg, respectively), twice daily, while those in group B inhaled 1 mg budesonide, twice daily. The patients in group C only received the routine treatment. The levels of IL-8 and IL-10 in the induced sputum, in addition to the blood gas levels and the lung function parameters were determined respectively prior to treatment and at a stable point, two weeks following the treatment.

### Arterial blood gas analysis

The partial pressure of oxygen in the arterial blood (PaO_2_) and the partial pressure of carbon dioxide in the arterial blood (PaCO_2_) for each patient were determined with 2 ml femoral arterial blood by an OPTI™ CCA-TS blood gas analyzer (OPTI Medical Systems, Inc., Roswell, GA, USA) under room air respiratory conditions.

### Lung function measurements

The percentage of forced expiratory volume in one second (FEV_1_)/predicted FEV_1_ (FEV_1%pred_) and the FEV_1_/forced vital capacity (FVC) ratio (FEV_1_/FVC) were measured three times successively with a Spirolab-II spirometer (MIR-Medical International Research, Rome, Italy) and the maximum values were selected.

### Induced sputum collection and handing

Prior to the experiment, each subject gargled with clear water and then inhaled 200 μg salbutamol. After 15 min, the patients inhaled ultrasonically nebulized 3% sodium chloride solution for 20 min. The patients were told to gargle with hydrogen peroxide and blow out any nasal discharge prior to each deep expectoration. The expectorated sputum was collected in an aseptic polypropylene box. The sputum, without the salivary component, was placed into a centrifuge tube and then 0.1% dithiothreitol (DTT) solution was added, according to a 1:1 quality/volume ratio. This was then placed on an oscillator and mixed well by shaking softly at room temperature. The sputum and DTT mixture was then placed in a 37° homeothermia swing bed for 15 min and centrifuged at 1,610 × g for 30 min. The supernate was subsequently obtained and reserved at −80°C, while the sediment was used for cytological analysis. Qualifying specimens contained <20% squamous cells and demonstrated a 50% cell survival rate.

### Determination of IL-8 and IL-10 levels in the induced sputum

The concentrations of IL-8 and IL-10 in the supernate of the induced sputum were determined by a double antibody sandwich enzyme-linked immunosorbent assay (ELISA). The reagents for the ELISA were purchased from Jingmei Biotechnology Co., Ltd. (Shenzhen, China), and were used in accordance with the manufacturer’s instructions. The optical density value at 450 nm was measured using a Bio-Rad 3550 ELISA reader (Bio-Rad, Hercules, CA, USA), and the IL-8 and IL-10 concentrations were calculated following the drawing of a standard curve.

### Statistics analysis

The experimental data, expressed as the mean ± standard deviation, were analyzed using SPSS l7.0 statistical software (SPSS, Inc., Chicago, IL, USA). Multi-interclass mean differential significance analysis was performed using analysis of variance, while self pre- and post-therapy values were compared using a paired t-test and correlation analysis was performed using a Pearson rectilinear analysis. The criteria of inspection were a=0.05 and P<0.05, where comparisons with these values were considered to indicate a statistically significant difference.

## Results

### Induced sputum IL-8 and IL-10 levels, lung function parameters and arterial blood gas levels prior to treatment

The induced sputum IL-8 level and the PaCO_2_ were significantly higher, while the induced sputum IL-10 level, the FEV_1%pred_, the FEV_1_/FVC and the PaO_2_ were significantly lower in the three COPD groups compared with those in the control group (all P<0.01). However, there were no significant differences among the three COPD groups (all P>0.05; Table I).

### Comparison of induced sputum IL-8 and IL-10 levels, lung function parameters and arterial blood gas levels following treatment

Compared with the pre-therapy values, the induced sputum IL-8 level and the PaCO_2_ were reduced, while the induced sputum IL-10 level, the FEV_1%pred_, the FEV_1_/FVC and the PaO_2_ were increased significantly (all P<0.01) in the three COPD groups. Furthermore, significant differences were apparent among the three groups (all P<0.01; Table II).

### Correlation analysis of IL-8 and IL-10 levels in the induced sputum with lung function parameters and arterial blood gas levels

The induced sputum IL-8 level was negatively correlated with FEV_1%pred_, FEV_1_/FVC and PaO_2_ (all P<0.001) and positively correlated with PaCO_2_. The IL-10 level was significantly positively correlated with FEV_1%pred_, FEV_1_/FVC and PaO_2_, and negatively correlated with PaCO_2_ (all P<0.001) in all patients prior to treatment (Table III).

## Discussion

IL-8 is a type of chemotactic factor that is important in the activation of neutrophilic granulocytes. It is synthesized in and secreted from alveolar macrophages, airway epithelial cells, neutrophils and lymphocytes. IL-8 is able to stimulate neutrophils to change shape, promoting degranulation and leading to the release of superoxide dismutase and lysosomal enzymes, which enhance the inflammatory reaction, resulting in airway and lung parenchyma inflammation and damage, accordingly. IL-8 is the predominant cytokine in the sputum of patients with COPD that induces neutrophil chemotaxis (15–17). IL-10 is synthesized in CD_4_^+^ or CD_8_^+^ T-lymphocytes, macrophages, monocytes, eosinophils and the airway epithelium. Macrophages and monocytes are the primary cells that generate endogenous IL-10. The function of IL-10 is to inhibit and terminate the inflammatory reaction, and to inhibit the synthesis and release of the proinflammatory cytokines. Therefore, IL-10 possesses anti-inflammatory effects (7,18).

The primary purpose of our study was to observe the expression of IL-8 and IL-10 in the induced sputum of patients with AECOPD complicated by CCP at high altitude. The results revealed that the induced sputum IL-8 level was significantly higher, whereas the induced sputum IL-10 level was significantly lower in the three COPD groups compared with the healthy control group, prior to treatment. It is possible that the increased levels of proinflammatory cytokines and decreased levels of anti-inflammatory cytokines in the airways of the patients with AECOPD complicated by CCP at high altitude may be the mechanism behind the enhanced inflammatory reaction in the airway. The results support our hypothesis and are in concurrence with the results from a previous study on COPD, conducted in flatland areas (8). CD_8_^+^ lymphocyte, macrophage and neutrophil levels have been demonstrated to increase in bronchial biopsy tissue, while IL-8, IL-6, tumor necrosis factor-α (TNF-α) and their soluble receptor levels have been demonstrated to increase significantly in the circulation and airducts of patients with COPD in flatland areas, indicating that persistent chronic inflammation is present in COPD (19). Furthermore, IL-8, leukotriene B4 (LTB4) and myeloperoxidase (MPO) levels in the sputum have been revealed to increase markedly in patients with AECOPD in flatland areas (20), as well as increasing in bronchial biopsy tissue and in the blood; however, the IL-10 concentration was observed to decrease significantly (10,11). The results of the present study revealed that the level of IL-8 in the induced sputum was significantly negatively correlated with FEV_1%pred_, FEV_1_/FVC and PaO_2_, and was significantly positively correlated with PaCO_2_. In addition, the level of IL-10 was observed to be significantly positively correlated with FEV_1%pred_, FEV_1_/FVC and PaO_2_, and significantly negatively correlated with PaCO_2_ in patients with AECOPD, complicated by CCP, at altitude. The results indicate that the IL-8 level in the sputum increased and the IL-10 level in the sputum decreased along with the extent of the airflow limitation in patients with AECOPD complicated by CCP at altitude. This was in concurrence with the results of a study conducted in flatland areas, which demonstrated that the more severe the airway obstruction in the patient with COPD in flatland areas, the higher the IL-8 concentration in the sputum (20 (MIR-Medical International Research, Rome, Italy)). The IL-8 concentration in the sputum has been observed to be negatively correlated with FEV_1_, while the IL-10 concentration in the sputum has been revealed to be positively correlated with FEV_1%pred_ and FEV_1_/FVC (8,11,21,22). All the previously mentioned results demonstrate that the increased secretion of the proinflammatory cytokine, IL-8, and the decreased secretion of the anti-inflammatory cytokine, IL-10, are involved in the pathogenesis of COPD, and change along with the severity of the airflow limitation. Thus, the inhibition of IL-8 and the promotion of IL-10 synthesis possess important clinical significance with regard to the control of airway inflammation in COPD and the improvement of the condition and lung function of the patients.

The results of our study indicate that the increase in IL-8 levels and reduction in IL-10 levels in the induced sputum of patients with AECOPD complicated by CCP at altitude, were more severe than those in flatland areas (10,11,19). This may be due to a number of reasons, such as the fact that altitude presents a special hypoxic environment. Under such conditions, patients with AECOPD complicated by CCP suffer from the combined effects of environmental hypoxia and an anoxic disease, and, therefore, the hypoxemia of patients at altitude is more severe than those in flatland areas. In addition, the patients in the current study suffered from COPD complicated by CCP; thus, their condition was more severe than that of patients solely with COPD in flatland areas. Furthermore, large differences in temperature and air quality may lead to repeated airway infection and acute exacerbation in patients with COPD. All these factors may have resulted in a more severe airway inflammation in patients with AECOPD complicated by CCP than in those in flatland areas.

In addition to assessing the expression of IL-8 and IL-10 in the induced sputum, the purpose of our study was to observe the intervention effects of an ICS combined with a LABA on the levels of IL-8 and IL-10 in the induced sputum of patients with AECOPD complicated by CCP at altitude. The results revealed that the induced sputum IL-8 level and the PaCO_2_ decreased significantly, while the induced sputum IL-10 level, FEV_1%pred_, FEV_1_/FVC and the PaO_2_ increased significantly in the three groups with COPD following treatment, compared with the levels measured pre-therapy. Furthermore, there were significant differences in the parameters among the three groups. It was demonstrated that, along with reductions in the IL-8 level and increases in the IL-10 level, the airway inflammation was reduced and the extent of the airflow limitation was improved following ICS treatment. In addition, ICS was able to effectively suppress the synthesis or secretion of the proinflammatory cytokine, IL-8, and promote the synthesis or secretion of the anti-inflammatory cytokine, IL-10.

The ICS/LABA combination inhalers produced the most efficacious results, followed by the sole use of ICS, while the conventional therapy was the least effective. This was consistent with our hypothesis and similar to the results from a previous study concerning COPD in flatland areas (23). The salmeterol/fluticasone propionate combination inhaler has been reported to significantly lessen airway hyper-inflammation in patients with COPD in flatland areas (24,25); the effect of the combination inhaler on inflammatory cells in COPD exceeded the effects of the inhalation or oral intake of corticosteroids alone. The regular inhalation of corticosteroids by patients with COPD in flatland areas is able to postpone the rapidity of the decline in lung function, reduce the episodes of acute exacerbation, ease disease severity, mitigate gasping and improve the quality of life, while the inhalation of an ICS combined with a LABA results in a more marked improvement in every parameter (26). The ability of salmeterol/fluticasone to reduce the acute exacerbation or severity of COPD has been demonstrated to be particularly notable (27). It has been revealed that the therapeutic effect of an ICS combined with a LABA surpasses the effect of an ICS used by itself. A possible mechanism for this is through the glucocorticosteroid mediating the upregulation of β_2_-adrenoceptor gene transcription, thus increasing the density and quantity of the β_2_-adrenoceptors on the cell surface and enhancing the sensitivity of the bronchial smooth muscle to β_2_-adrenoceptor agonists. In addition, the LABA may accelerate the translocation of glucocorticoid receptors to the leucocytic nucleus, promoting glucocorticoid-sensitive gene transcription and enhancing its anti-inflammatory activity. Glucocorticoids act at the airway inflammation, while LABAs act at the smooth muscle of the airway. Combining a glucocorticoid with a LABA produces a synergistic effect that may effectively depress airway inflammation and improve corticosteroid sensitivity (28). This represents a key intervention for preventing and treating AECOPD, in addition to preventing its development by inhibiting the non-specific airway inflammation or interrupting the cytokine-mediated inflammatory pathway. A study by Patel *et al*(29) revealed that the airway epithelium of patients with COPD displayed a significant cytokine response following inflammatory stimulation, and that there was a marked reduction in IL-8 secretion following the administration of an ICS. Armstrong *et al*(30) reported that the activation of p38 mitogen-activated protein kinase (MAPK) decreased pulmonary alveolar macrophage sensitivity to glucocorticoids, while a p38 MAPK inhibitor combined with a glucocorticoid was able to strengthen the anti-inflammatory action by suppressing cytokine secretion.

The current study suggests that ICS/LABA combination inhalers may effectively control airway inflammation in patients with AECOPD complicated by CCP, abate airflow limitations and improve the condition of the patients. ICS/LABA combination inhalers are a safe and valid approach for treating COPD complicated by CCP and their effects surpass those of simple ICS therapy.

Further studies are required to investigate the reason why levels of IL-8 increase and the levels of IL-10 decrease more markedly in the sputum of patients with AECOPD complicated by CCP at altitude than in those in flatland areas. In addition, we have planned a comparative study comparing simple COPD at altitude with that in flatland areas.

## Figures and Tables

**Table I:** Comparison of IL-8 and IL-10 levels in the induced sputum, lung function parameters and arterial blood gas levels prior to treatment in the four groups.

Group	IL-8 (pg/ml)	IL-10 (pg/ml)	FEV_1%pred_	FEV_1_/FVC (%)	PaO_2_ (mmHg)	PaCO_2_ (mmHg)
A	408.56±43.57^a^	34.57±7.33^a^	31.85±5.72^a^	37.12±6.86^a^	32.84±5.52^a^	58.96±6.21^a^
B	402.15±42.85^a^	34.80±7.08^a^	32.36±6.28^a^	37.35±6.34^a^	32.62±6.09^a^	59.13±6.53^a^
C	395.67±42.60^a^	35.63±7.14^a^	32.34±6.25^a^	36.97±6.13^a^	33.16±5.45^a^	59.47±6.09^a^
D	132.20±12.63	67.33±6.35	71.15±6.53	75.44±6.81	65.36±5.33	28.62±4.05

Data are presented as the mean ± standard deviation. Group A, salmeterol/fluticasone treatment group; Group B, budesonide treatment group; Group C, routine treatment group; Group D, control group.

a P<0.01 compared with group D.

IL, interleukin; FEV_1_, percentage of forced expiratory volume in one second; FEV_1%pred_, predicted FEV_1_; FVC, forced vital capacity; PaO_2_, partial pressure of oxygen in arterial blood; PaCO_2_, partial pressure of carbon dioxide in arterial blood.

**Table II:** Comparison of IL-8 and IL-10 levels in the induced sputum, lung function parameters and arterial blood gas levels following treatment in the three groups.

Group	IL-8 (pg/ml)	IL-10 (pg/ml)	FEV_1%pred_	FEV_1_/FVC (%)	PaO_2_ (mmHg)	PaCO_2_ (mmHg)
A	181.50±33.72^a^^b^	54.22±6.54^a^^b^	45.03±6.36^a^^b^	47.88±6.30^a^^b^	54.52±6.27^a^^b^	41.43±4.28^a^^b^
B	262.63±34.35^a^^c^	47.19±7.25^a^^c^	40.12±4.27^a^^c^	45.24±5.30^a^^c^	49.75±5.46^a^^c^	45.77±5.23^a^^c^
C	307.55±33.60^a^	40.66±6.42^a^	36.53±5.22^a^	40.94±5.72^a^	45.61±4.32^a^	50.47±6.07^a^

Data are presented as the mean ± standard deviation. Group A, salmeterol/fluticasone treatment group; Group B, budesonide treatment group; Group C, routine treatment group.

a P<0.01 compared with the value prior to treatment;

b P<0.01 compared with groups B and C;

c P<0.01 compared with group C.

IL, interleukin; FEV_1_, percentage of forced expiratory volume in one second; FEV_1%pred_, predicted FEV_1_; FVC, forced vital capacity; PaO_2_, partial pressure of oxygen in arterial blood; PaCO_2_, partial pressure of carbon dioxide in arterial blood.

**Table III:** Correlation analysis of induced sputum IL-8 and IL-10 levels, lung function parameters and arterial blood gas levels prior to treatment in 186 patients

	FEV_1%pred_		FEV_1_/FVC		PaO_2_		PaCO_2_	
				
Parameters	r value	P-value	r value	P-value	r value	P-value	r value	P-value
IL-8	−0.726	<0.001	−0.667	<0.001	−0.688	<0.001	0.658	<0.001
IL-10	0.673	<0.001	0.648	<0.001	0.667	<0.001	−0.599	<0.001

IL, interleukin; FEV_1_, percentage of forced expiratory volume in one second; FEV_1%pred_, predicted FEV_1_; FVC, forced vital capacity; PaO_2_, partial pressure of oxygen in arterial blood; PaCO_2_, partial pressure of carbon dioxide in arterial blood.
